# Quantitative dopamine transporter imaging assessment in Parkinson's disease patients carrying GBA gene mutations compared with idiopathic PD patients: A case‐control study

**DOI:** 10.1002/brb3.3060

**Published:** 2023-06-01

**Authors:** Sara Grisanti, Alessandro Fraternali, Francesco Cavallieri, Valentina Fioravanti, Massimiliano Casali, Giulia Toschi, Laura Ferri, Rossella Sabadini, Marialuisa Zedde, Gaetano Salomone, Manuela Napoli, Rosario Pascarella, Valeria Ferrari, Maria Scarano, Giuseppe Biagini, Augusto Scaglioni, Elena Moro, Annibale Versari, Franco Valzania

**Affiliations:** ^1^ Clinical and Experimental Medicine PhD Program University of Modena and Reggio Emilia Modena Italy; ^2^ Nuclear Medicine Unit Azienda USL‐IRCCS di Reggio Emilia Reggio Emilia Italy; ^3^ Neurology Unit, Neuromotor and Rehabilitation Department Azienda USL‐IRCCS di Reggio Emilia Reggio Emilia Italy; ^4^ Department of Neuroscience, Imaging and Clinical Sciences “G. D'Annunzio” University of Chieti‐Pescara Chieti Italy; ^5^ Neuroradiology Service, Department of Diagnostic Imaging and Laboratory Medicine Azienda USL‐IRCCS di Reggio Emilia Reggio Emilia Italy; ^6^ Dipartimento di Cure Primarie Azienda Unità Sanitaria Locale (AUSL) Modena Modena Italy; ^7^ Department of Biomedical, Metabolic and Neural Sciences University of Modena and Reggio Emilia Modena Italy; ^8^ Neurorehabilitation Unit Center Fondazione Don Gnocchi Parma Italy; ^9^ Grenoble Alpes University, Division of Neurology, Centre Hospitalier Universitaire de Grenoble Grenoble Institute of Neuroscience Grenoble France

**Keywords:** DaTSCAN, DaTQUANT, GBA, Glucocerebrosidase, genetic, I‐123 FPCIT SPECT, Parkinson's Disease, PD

## Abstract

**Background:**

Genetic risk factors impact around 15% of Parkinson's disease (PD) patients and at least 23 variants have been identified including Glucocerebrosidase (GBA) gene variants. Using different clinical and instrumental qualitative‐based data, various studies have been published on GBA‐PD cohorts which suggested possible differences in dopaminergic nigrostriatal denervation pattern, particularly in caudate and putamen nuclei.

**Methods:**

This retrospective study included two consecutive homogenous cohorts of GBA‐PD and idiopathic (I‐PD) patients. Each consecutive GBA‐PD patient has been matched with a 1:1 pairing method with a consecutive I‐PD subject according to age, age at disease onset, sex, Hoehn & Yahr (H&Y) staging scale and comorbidity level (CCI). Semiquantitative volumetric data by the DaTQUANT^TM^ software integrated in the DaTSCAN exam performed at time of the diagnosis (SPECT imaging performed according to current guidelines of I‐123 FPCIT SPECT imaging) were extrapolated. Bilateral specific binding ratios (SBR) at putamen and caudate levels were calculated, using the occipital lobes uptake. The Mann–Whitney test was performed to compare the two cohorts while the Spearman's test was used to find correlations between motor and volumetric data in each group. Bonferroni correction was used to account for multiple comparisons.

**Results:**

Two cohorts of 25 patients each (GBA‐PD and I‐PD), were included. By comparing GBA‐PD and I‐PD patients, lower SBR values were found in the most affected anterior putamen and left caudate of the GBA‐PD cohort. Furthermore, in the GBA‐PD cohort the SBR of the most affected posterior putamen negatively correlated with the H&Y scale. However, none of these differences or correlations remained significant after Bonferroni correction for multiple comparisons.

**Conclusions:**

We observed differences in SBR values in GBA‐PD patients compared with I‐PD. However, these differences were no longer significant after Bonferroni multiple comparisons correction highlighting the need for larger, longitudinal studies.

## Graphical abstract

Two homogenous cohorts of GBA‐PD and idiopathic PD (I‐PD) patients were investigated by single photon emission computed tomography using Ioflupane I123 and quantified by DaTQUANT(TM) software to evaluate changes correlated with different clinical variables. This study revealed differences in dopaminergic denervation, especially in the left caudate and anterior putamen of GBA‐PD patients.

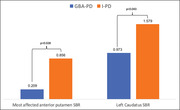

## 1 INTRODUCTION

Parkinson's disease (PD) is a neurodegenerative disease with different motor and nonmotor symptoms. Based on clinical and prodromal criteria, various PD subtypes have been identified (Poewe et al., 2017). Genetic risk factors impact around 15% of PD patients (Berg et al., 2021) and at least 23 variants have been identified, the most common ones located in Leucine‐rich repeat kinase 2 (LRKK2) and Glucocerebrosidase (GBA) genes (Berg et al., 2021; Poewe et al., 2017). When compared to idiopathic PD (I‐PD), GBA‐PD patients might manifest a different PD phenotype, with a relative earlier disease onset, greater cognitive impairment and, overall, a more aggressive disease progression (Omer et al., 2022; Petrucci et al., 2020). Using different clinical and instrumental qualitative‐based data, various studies have been published on GBA‐PD cohorts, which suggested interesting differences in dopaminergic deficit, particularly in caudate and putamen nuclei (Filippi et al., 2022). However, due to the complexity in gene mutation screening and difficulties to obtain homogeneous cohorts, results and observations were often inconsistent also because not comparable (Filippi et al., 2022; Omer et al., 2022). Here we report the results of a cohort analysis based on a semiquantitative volumetric acquisition software integrated in the DaTSCAN exam, applied to a group of GBA‐PD patients, and compared to a matched I‐PD cohort.

## 2 METHODS

This retrospective study included two consecutive cohorts of GBA‐PD and I‐PD patients. The genetic profile was obtained by testing the patients for the presence of 11 pathogenic or likely pathogenic LRKK2 variants and GBA sequences. If negative, a next‐generation sequencing panel targeting 68 genes involved in PD was performed. Each consecutive GBA‐PD patient has been matched with a 1:1 pairing method with a consecutive I‐PD subject according to age, age at disease onset, sex and comorbidity level (CCI). In particular, consecutive GBA‐PD patients with available clinical and Datscan data from an original pull of 50 GBA‐PD patients were included in the study. For each GBA‐PD patient, a 1:1 match has been performed searching from a pull of 400 consecutive I‐PD subjects. In detailed, the first anonymized patient in a list in random order with matched sex, age, age at disease onset (with a tolerance interval of 2 years), comorbidity index (CCI) and available DaTSCAN data has been selected. During the pairing method, no other clinical or instrumental data has been considered except the one already mentioned.

For each patient the following clinical items have been collected: H&Y scale, motor phenotype (akineto‐rigid, tremor‐dominant), body side of motor symptoms’ onset, disease duration, Levodopa Equivalent Daily Dose (LEDD). Semiquantitative volumetric data by the DaTQUANT software (GE Healthcare) integrated in the DaTSCAN exam performed at time of the diagnosis were extrapolated. Bilateral specific binding ratios (SBR) at putamen and caudate levels were calculated, using the occipital lobes uptake as the background reference region, together with bilateral putamen/caudate ratios and putamen/caudate asymmetries. Moreover, putamen and caudate SBR in the most affected and least affected hemisphere were also computed (i.e., the lower/higher value between left and right hemisphere) as previously reported (Filippi et al., 2022). The Mann–Whitney test was performed to compare the two cohorts while the Spearman's test was used to find correlations between motor and volumetric data in each group. A *p* value < .05 was considered significant. Bonferroni correction was used to account for multiple comparisons. Statistical analysis was performed using the IBM SPSS Statistics for Windows version 20.0 (IBM, Armonk, NY, USA). This study was approved by the Ethical Committee of the Area Vasta Emilia Nord (*n* = 536/2021), Italy and written informed consent was obtained from participants.

## 3 RESULTS

**FIGURE 1:**
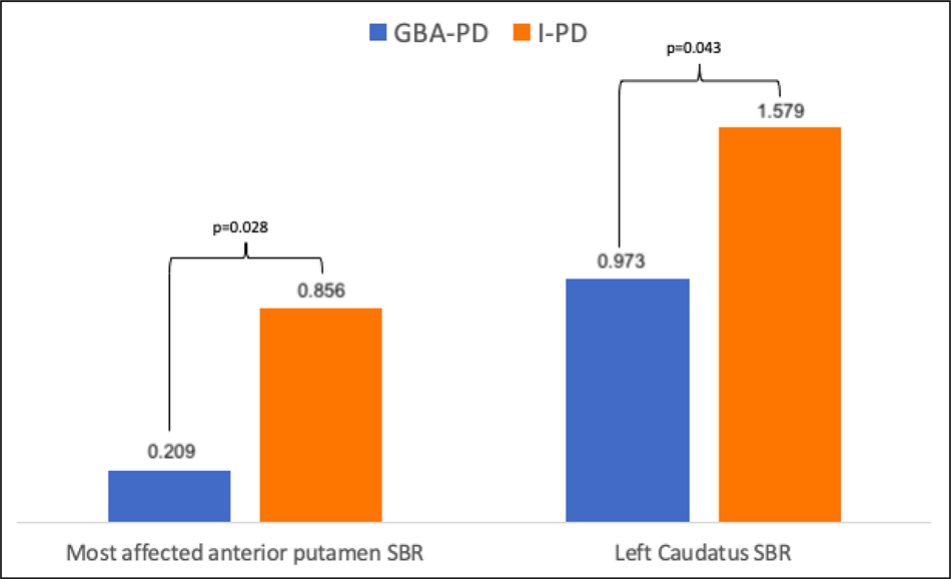
Significant differences in volumetric parameters. Bar plot reporting the two significant comparisons between GBA‐PD and I‐PD in terms of volumetric data (SBR of most affected anterior putamen and SBR of left caudate) from DaTQUANT analyses. Volumetric row data and *p* value of the comparisons are reported.

Two cohorts of 25 patients each (GBA‐PD and I‐PD), statistically homogeneous in terms of sex, age at disease onset, disease duration, Charlson Comorbidity Index (CCI), motor phenotype, Hoehn & Yahr (H&Y) staging scale, and side of disease onset were recruited for the study (Table 1). By comparing GBA‐PD and I‐PD patients, statistically significant lower values of specific binding ratio (SBR) were found in the most affected anterior putamen (*p* = .028, Mann–Whitney test) and left caudate (*p* = .043) of the GBA‐PD cohort compared with the I‐PD cohort (Figure 1). Furthermore, in the GBA‐PD cohort the SBR of the most affected posterior putamen negatively correlated with the H&Y scale (ρ = −0.568, *p* = .003). In terms of correlation in the I‐PD group, the SBR of the most affected caudate inversely correlated with the LEDD (ρ = −0.460, *p* = .021). However, none of these differences or correlations remained significant after Bonferroni correction for multiple comparisons.

**TABLE 1:** Clinical and volumetric analyses

	All (*n* = 50)	GBA‐PD (*n* = 25)	I‐PD (*n* = 25)	*p* Value
Variable—clinical	No. (%); mean [SD]; median {range}			
Age	65.18 [8.98]; 63 {50–82}	65.36 [8.97]; 63 {50–82}	65.00 [9.18]; 63 {50–82}	.86
Age at diagnosis	58.26 [9.46]; 57 {43–75}	58.6 [9.54]; 57 {43–75}	57.90 [9.57]; 55 {43–74}	.81
CCI	2.78 [1.83]; 2 {0–8}	2.60 [1.47]; 2 {0–5}	2.96 [2.15]; 3 {0–8}	.84
LEDD	688.06 [418.91]; 622 {0–2300}	678.72 [385.70]; 634 {0–1521}	697.40 [457.53]; 550 {80–2300}	.79
H&Y	2.33 [0.52]; 2.5 {1–4}	2.26 [0.36]; 2.00 {2–3}	2.40 [0.65]; 2.50 {1–4}	.23
Disease duration	6.92 [3.67]; 6 {2–15}	6.72 [3.89]; 6 {2–14}	7.12 [3.52]; 6 {3–15}	.63
Sex, male	30/50 (60%)	15/25 (60%)	15/25 (60%)	1
Motor phenotype, akineto‐rigid	35/50 (70%)	19/25 (76%)	16/25 (64%)	.36
PD onset side, right	29/50 (58%)	14/25 (56%)	15/25 (60%)	.78

	All (*n* = 50)	GBA‐PD (*n* = 25)	I‐PD (*n* = 25)	*p* Value	DaTQUANT normal values for subjects aged 65 years
Variable—DaTQUANTTM	No. (%); mean [SD]; median {range}				
Striatum L SBR	1.22 [0.77]; 1.33 {0.01–3.41}	1.14 [0.82]; 1.10 {0.10–3.41}	1.29 [0.72]; 1.39 {0.01–3.13}	.39	2.47 [0.42]
Striatum R SBR	1.21 [0.76]; 1.35 {0.07–2.67}	1.22 [0.79]; 1.33 {0.09–2.67}	1.19 [0.74]; 1.44 {0.07–2.38}	.81	2.46 [0.40]
Most affected striatum SBR	1.06 [0.75]; 1.27 {0.01–2.67}	1.22 [0.79]; 1.34 {0.09–2.67}	0.90 [0.69]; 1.13 {0.01–2.38}	.18	N/A
Least affected striatum SBR	1.53 [0.64]; 1.49 {0.11–3.41}	1.47 [0.69]; 1.47 {0.11–3.41}	1.58 [0.59]; 1.56 {0.23–3.13}	.32	N/A
Caudate L SBR	1.29 [0.92]; 1.44 {0.002–4.47}	1.10 [1.05]; 0.97 {0.002–4.47}	1.48 [0.74]; 1.58 {0.21–2.70}	.043*	2.70 [0.50]
Caudate R SBR	1.21 [0.99]; 1.35 {0.01–3.67}	1.25 [1.07], 1.48 {0.01–3.67}	1.18 [0.94]; 1.12 {0.15–2.55}	.87	2.66 [0.48]
Most affected caudate SBR	0.78 [0.82]; 0.27 {0.002–3.67}	0.73 [0.91]; 0.21 {0.002–3.67}	0.83 [0.74]; 0.30 {0.15–2.20}	.11	N/A
Least affected caudate SBR	1.72 [0.84]; 1.76 {0.21–4.47}	1.62 [1.01]; 1.60 {0.21–4.47}	1.82 [0.64]; 1.81 {0.24–2.70}	.22	N/A
Putamen L SBR	1.22 [1.06]; 1.06 {0.03–4.78}	1.21 [1.17]; 0.95 {0.03–4.78}	1.22 [0.95]; 1.18 {0.10–4.72}	.43	2.36 [0.41]
Putamen R SBR	1.21 [0.88]; 1.15 {0.001–4.79}	1.21 [0.77]; 1.14 {0.08–3.38}	1.22 [0.99]; 1.20 {0.001–4.79}	.96	2.38 [0.39]
Most affected putamen SBR	0.87[0.89]; 0.89 {0.001–4.72}	0.83 [0.79]; 0.89 {0.03–3.38}	0.91 [0.99]; 0.90 {0.001–4.72}	.65	N/A
Least affected putamen SBR	1.56 [0.93]; 1.28 {0.099–4.79}	1.59 [1.02]; 1.24 {0.28–4.78}	1.53 [0.85]; 1.31 {0.10–4.79}	.66	N/A
Anterior putamen L SBR	1.03 [0.66]; 1.13 {0.02–2.46}	0.98 [0.69]; 1.01 {0.02–2.46}	1.08 [0.64]; 1.29 {0.12–2.04}	.43	2.49 [0.43]
Anterior putamen R SBR	1.12 [0.76]; 1.23 {0.02–3.46}	0.999 [0.87]; 0.999 {0.03–3.46}	1.23 [0.61]; 1.39 {0.02–1.99}	.12	2.51 [0.42]
Most affected anterior putamen SBR	0.65 [0.58]; 0.35 {0.02–1.95}	0.50 [0.53]; 0.21 {0.02–1.95}	0.79 [0.60]; 0.86 {0.02–1.87}	.028*	N/A
Least affected anterior putamen SBR	1.5 [0.55]; 1.44 {0.13–3.46}	1.48 [0.68]; 1.37 {0.13–3.46}	1.52 [0.40]; 1.49 {0.57–2.04}	.49	N/A
Posterior putamen L SBR	0.93 [0.84]; 0.78 {0.01–4.01}	0.70 [0.41]; 0.65 {0.13–1.59}	1.16 [1.08]; 0.93 {0.01–4.01}	.09	2.09 [0.41]
Posterior putamen R SBR	1.02 [0.96]; 0.83 {0.05–4.90}	1.08 [1.01]; 0.86 {0.07–4.90}	0.97 [0.92]; 0.82 {0.05–4.42}	.71	2.10 [0.38]
Most affected posterior putamen SBR	0.64 [0.64]; 0.59 {0.01–4.00}	0.57 [0.41]; 0.54 {0.07–1.59}	0.71 [0.82]; 0.70 {0.01–4.01}	.90	N/A
Least affected posterior putamen SBR	1.31 [0.99]; 1.03 {0.11–4.90}	1.21 [0.94]; 0.97 {0.37–4.90}	1.41 [1.05]; 1.07 {0.11–4.42}	.28	N/A
Striatum asym	1.26 [1.10]; 1.04 {0.02–5.80}	1.24 [0.97]; 1.09 {0.04–4.82}	1.28 [1.24]; 0.99 {0.02–5.80}	.87	0.03 [0.02]
Caudate asym	1.37 [1.17]; 1.08 {0.08–5.64}	1.44 [1.23]; 1.25 {0.14–5.64}	1.30 [1.12]; 0.95 {0.08–3.42}	.46	0.05 [0.04]
Putamen asym	1.02 [0.82]; 0.79 {0.00–3.55}	1.15 [0.93]; 0.94 {0.0002–3.55}	0.90 [0.68]; 0.70 {0.19–2.48}	.33	0.03 [0.03]
Putamen L ratio	0.73 [0.24]; 0.79 {0.07–1.11}	0.76 [0.24]; 0.79 {0.07–1.11}	0.69 [0.24]; 0.78 {0.08–0.94}	.27	0.91 [0.06]
Putamen R ratio	0.76 [0.23]; 0.79 {0.01–1.16}	0.81 [0.20]; 0.83 {0.08–1.16}	0.70 [0.25]; 0.76 {0.01–0.99}	.09	0.92 [0.06]

No. (%); mean [SD]; median {range} according to statistical analysis performed (% feature on overall, mean, standard deviation, median, range). On the right the *p* value from GBA‐PD VS I‐PD comparison is reported. *Significant values.

Abbreviations: asym: asymmetry; CCI: Charlson Comorbidity Index; H&Y: Hoehn & Yahr; LEDD: Levodopa Equivalent Daily Dose N/A: not available; PD: Parkinson's disease; SBR: specific binding ratio.

## 4 DISCUSSION

This study reports differences of specific volumetric parameters between two homogenous cohorts of GBA‐PD and I‐PD subjects that however were not confirmed after Bonferroni correction for multiple comparisons. In particular, the SBR of the most affected anterior putamen and the left caudate were more impaired in the GBA‐PD than in I‐PD. These findings are in line with a previous study that showed a more pronounced dopaminergic dysfunction in a large GBA‐PD cohort compared with I‐PD, probably resulting from a more severe motor impairment in the GBA‐PD cohort (Cilia et al., 2016; Lee et al., 2021; McNeill et al., 2013; Simuni et al., 2020). In contrast, in our study the disease severity was comparable between the two cohorts to suggest that the GBA mutations themselves could influence the degree of dopaminergic dysfunction, at least in some parameters. A correlation between dopamine depletion in the anterior putamen and both early development of wearing‐off and dementia has been previously reported (Chung et al., 2020). Bearing in mind that motor and nonmotor fluctuations, as well as the cognitive decline develop earlier and with a more pronounced severity in GBA‐PD patients than in I‐PD, we might assume that the higher dopaminergic dysfunction in the anterior putamen found in our GBA‐PD partly explains the clinical differences and disease's progression between GBA‐PD and I‐PD (Petrucci et al., 2020). Unfortunately, in our study data about cognitive performance and motor and nonmotor fluctuations were not included now, representing a limitation of the study. Concerning the reduction of SBR in left caudate in GBA‐PD, a significant relationship between the loss of dopaminergic input and the expression of a cognitive‐related disease network in PD patients has been already reported (Niethammer et al., 2013). Furthermore, we found a negative correlation in the GBA‐PD between the SBR of the most affected posterior putamen and the H&Y scale. This is not surprising, considering the already well reported negative correlation between baseline DAT binding in the posterior putamen and PD motor severity (Palermo et al., 2021). However, this correlation was not confirmed in the I‐PD cohort, and in the just mentioned study the PD‐related genetic mutations in patients analyzed were not specified. A possible explanation for this discrepancy could be the reduced low sample size in our case, or the similar severity found in the two groups evaluated by the H&Y score, which we know to be a very gross parameter unable to guarantee that GBA‐PD patients will not further progress in the disease when challenged with more refined scales, such as the MDS‐UPDRS, which we plan to integrate in future longitudinal analyses. Furthermore, it cannot be excluded that this region of the putamen could also correlate with other components of the disease, already expressed in GBA and not in I‐PD. Even if this study has several limitations, including the low sample size and the lack of additional detailed quantification of motor symptoms and cognitive impairment, it represents the first to use specific semiquantitative software analyses over Datscan acquisitions in two homogeneous cohorts of GBA‐PD and I‐PD to reveal quantitative differences in dopaminergic dysfunction. This may suggest that the nigrostriatal system denervation may differ in GBA‐PD, especially in the most affected anterior putamen and left caudate. However, these differences were not confirmed after Bonferroni correction for multiple comparisons, highlighting the need for larger, longitudinal studies to further define the specific correlations between the posterior putamen and the cardinal features of GBA‐PD.

## AUTHOR CONTRIBUTIONS

Research conception: S.G. A.F., F.C., A.V., and F.V. Research organization: S.G., A.F., F.C., V.F., G.T., M.Z., M.N., R.P., G.B., E.M., A.V., F.V. Research execution: All authors. Design of analysis methods: S.G., A.F., F.C., F.V. Analysis execution: S.G., A.F., F.C. Review and critique of analysis: R.S., M.Z., G.B., A.S., E.M., A.V., F.V. Manuscript writing: S.G., A.F., F.C., V.F., E.M., F.V. Manuscript review and editing: S.G., A.F., F.C., V.F., M.C., G.T., L.F., R.S., M.Z., G.S., M.N., R.P., V.F., M.S., G.B., A.S., E.M., A.V., F.V. All authors approve the final version for publication.

## CONFLICT OF INTEREST STATEMENT

F. Cavallieri received personal fees from Zambon outside the submitted work. E. Moro has received honoraria from Abbott, Medtronic, Kyowa, and Newronika for consulting and lecturing and received an educational grant from Boston Scientific. The other authors report no disclosures.

### PEER REVIEW

The peer review history for this article is available at https://publons.com/publon/10.1002/brb3.3060

## Data Availability

The data that support the findings of this study are available from the corresponding author upon reasonable request.
